# Healthcare costs associated with progressive diabetic retinopathy among National Health Insurance enrollees in Taiwan, 2000-2004

**DOI:** 10.1186/1472-6963-10-136

**Published:** 2010-05-26

**Authors:** Lin-Chung Woung, Ching-Yao Tsai, Hsin-Kai Chou, Ming-Tsu Tsai, Wei-Her Tsai, Pesus Chou, Shih-Tsuo Shen

**Affiliations:** 1 Department of Ophthalmology, Taipei City Hospital, Taipei, Taiwan; 2 Department of Information Management, National Chung Cheng University, Chayi, Taiwan; 3 Department of Health Care Management, National Taipei College of Nursing, Taipei, Taiwan; 4 Taipei County Hospital, Taipei, Taiwan; 5 Chihlee Institute of Technology, Taipei, Taiwan; 6 Community Medicine Research Center and Institute of Public Health, National Yang-Ming University, Taipei, Taiwan; 7 Department of Ophthalmology, College of Medicine, National Taiwan University, Taipei, Taiwan

## Abstract

**Background:**

Diabetic retinopathy is one of the most common microvascular complications of diabetes and one of the major causes of adult visual impairment in national surveys in Taiwan. This study aimed to identify the healthcare costs of Taiwan's National Health Insurance program on behalf of diabetic patients with stable or progressive retinopathy.

**Methods:**

A retrospective cohort study was conducted with 4,988 medication-using diabetic retinopathy subjects ≥ 40 years of age under National Health Insurance Program coverage between 2000 and 2004. Study cohort subjects were recorded as having diabetic retinopathy according to ICD-9-CM codes. States of diabetic retinopathy were strategically divided into stable and progressive categories according to subjects' conditions at follow-up in 2004. Expenditures were calculated and compared for the years 2000 and 2004.

**Results:**

During the 4-year follow-up (2000 through 2004), 4,116 subjects (82.5%) of 4,988 diabetic subjects were in the stable category, and 872 (17.5%) were in the progressive category. Average costs of those in the normal category increased by US $48 from US $1921 in 2000 to US $1969 in 2004 (p = 0.594), whereas costs for those progressing from normal to non-proliferative diabetic retinopathy (NPDR) or proliferative diabetic retinopathy (PDR) increased by US $1760, from US $1566 in 2000 to US $3326 in 2004 (p < 0.001). The PDR category had the highest average costs at US $3632 in 2000. The NPDR-to-PDR category experienced the greatest increase in costs at US $3482, from US $2723 in 2000 to US $6204 in 2004 (p = 0.042), and the greatest percentage of increase at 2.3% (2.2% when adjusted by comparing to normal category).

**Conclusions:**

This large-scale longitudinal study provides evidence that increased healthcare costs are associated with progressive diabetic retinopathy among diabetic NHI enrollees in Taiwan.

## Background

Diabetes mellitus is a common, chronic disease among adults, with a worldwide prevalence estimated at 4% in 1995 and projected to rise to 5.4% by the year 2025 [1]. In Taiwan, diabetes is one of the most common chronic diseases, with a prevalence rate among adults estimated at between 4.9 and 8.0% from 1985 to 1995 [2-4]. As a result, both the demand for and the costs of medical care for diabetic patients is increasing [5].

Healthcare costs for diabetics are more than double the costs of those without diabetes in the United States [6] and the occurrence of major diabetes-related complications in type 2 diabetics patients is associated with increased average medical costs annually [7,8]. Diabetic retinopathy is one of the most common microvascular complications of diabetes and is one of the major causes of adult visual impairment in national surveys conducted in both Taiwan [9] and the United States [10].

Increased healthcare costs attract the attention of policy makers seeking strategies to reduce medical costs. Despite the enormity of the problem, nationwide data about the costs of treating diabetic patients with diabetic retinopathy are still limited. A common source of administrative data is health insurance claims data associated with private or public health insurance programs. In Taiwan, 96% of all residents have been enrolled in the National Health Insurance (NHI) program since 1996. In this study, we used these administrative claims data to identify the costs of the NHI program paid on behalf of diabetic patients with either stable or progressive retinopathy.

## Methods

### Data source and study population

In Taiwan, the National Health Insurance (NHI) Program began on March 1, 1995. As of 2007, 22.6 million of Taiwan's 23 million persons were enrolled in this program. Therefore, the NHI Program has accumulated 23.8 million administrative and claims records. In order to respond to current and emerging issues rapidly and effectively, the National Health Research Institutes (NHRI) cooperated with the NHI Bureau (NHIB) to establish and maintain an NHI research database. The NHRI safeguards the privacy and confidentiality of subjects and routinely transfers insurance health data from the NHIB to health researchers for analysis, with the aim of improving the health of Taiwan's citizens. Data analysed in this study were also retrieved from the National Health Insurance Research Database. Access to research data has been approved by the Review Committee of the NHRI. The NHRI provides a unique validated database of medical claims from 200,432 random subjects, representing about 1% of the population, for use in health insurance studies. These data consisted of inpatient and ambulatory care records, as well as registration files, and resulted in the establishment of an academic, retrospective cohort group. This academic cohort database has been tested and no notable differences were found in age, sex, or medical costs for all enrollees [11]. Likewise, several studies have been conducted to evaluate the prevalence, incidence, and other associated factors of various diseases using the NHI research database [12-14].

Since 1999, the NHRI database has used the International Classification of Diseases, 9th Revision, Clinical Modification (ICD-9-CM) for outpatient and inpatient diagnostic coding. By searching these electronic records and pharmacy prescriptions in similar fashion to a previous study [15], individuals were identified who used medications typically prescribed for diabetes (insulin or oral hypoglycemic agents) during the period of observation. For the yearly data set in both 2000 and 2004, subjects in the study cohort were recorded as having diabetic retinopathy if we found the following ICD-9-CM codes during the search of administrative or outpatient data: 362.0 (non-proliferative diabetic retinopathy, NPDR), and 362.0 (proliferative diabetic retinopathy, PDR). The normal status was recorded when no evidence of diabetic retinopathy was found. The states of diabetic retinopathy were strategically divided into stable and progressive categories according to the subjects' conditions at follow-up in 2004.

### Estimation of medical costs for diabetes

All estimates of medical costs, including copayments, were calculated in New Taiwan dollars (NT$). Fee-for-service costs included outpatient and inpatient care, prescription drugs, and laboratory fees. Costs represented healthcare expenditures for an individual for the whole year, not focusing exclusively on those associated with diabetes or diabetic retinopathy. Outpatient services included outpatient visits, emergency department visits, end-stage renal dialysis, preventive services, dental care, home healthcare, and traditional Chinese medicine treatments. Inpatient care costs were calculated over a one-year period in both 2000 and 2004. The costs in this study did not include services that might have been provided but were not covered by NHI. Indirect costs of diabetes such as lost productivity due to disability or premature death were not estimated.

Excess costs associated with changes in diabetic retinopathy were measured and compared as average annual costs for 2000 and 2004. Changes in annual costs associated with diabetic retinopathy were estimated by comparing mean medical costs in 2000 and 2004 for the two main categories of stable and progressive retinal status. These included two subcategories in the stable group (normal-to-normal, NPDR-to-NPDR, and PDR-to-PDR), and three subcategories in the progressive group (normal-to-NPDR and PDR, NPDR-to-PDR).

### Statistical analysis

The statistical significance of differences identified in mean costs was evaluated using the paired *t *test because claim years for the same individual were assumed to be inherently related.

## Results

In our study population, the number of medication-using diabetic subjects ≥ 40 years of age was 4,988 under NHI coverage in 2000. The expenditures of all subjects were calculated and compared for the years 2000 and 2004. The characteristics of these subjects are presented in Table 1. Among all subjects, 2,642 (53.0%) were female, and 2,192 (44.0%) were considered elderly (age 65 and older).

**Table 1:** Characteristics of study population in 2000.

Variables	Number of subjects	%
**Gender**		
**Males**	2346	47.0
**Females**	2642	53.0
**Age (years)**		
**40-54**	1313	26.3
**55-64**	1483	29.7
≧65	2192	44.0
**Total**	4988	100.0

### Changing status of diabetic retinopathy

Table 2 shows 4,116 (82.5%) of 4,988 diabetic subjects who were in the stable category, and 872 (17.5%) who were in the progressive category during the 4-year follow-up. Among those 4,116 stable individuals, 91.6% subjects were classified as normal, 5.1% as NPDR, and 3.3% as PDR. Among 872 in the progressive category, 93.5% had no diabetic retinopathy in 2000, but had progressed to NPDR or PDR by 2004; 6.5% had NPDR in 2000 and progressed to PDR in 2004.

**Table 2:** Medical Costs for Diabetic Retinopathy between 2000 and 2004.

Group	Baseline/Final Status	n	%	Year	Total Cost*	2004/2000 ratio (adjusted ratio)	Excess Cost*	P Value**
**Stable**								
	**Normal/Normal**	3772	75.6	2000	62, 218	1.02 (reference = 1)	1, 543	0.594
				2004	63, 761			
	**NPDR/NPDR**	208	4.2	2000	75, 506	1.38 (1.35)	29, 316	0.086
				2004	104, 822			
	**PDR/PDR**	136	2.7	2000	117, 630	1.55 (1.50)	64, 123	0.036
				2004	181, 753			

**Progressive**								
	**Normal/NPDR or PDR**	815	16.4	2000	50, 712	2.12 (2.07)	57, 020	<0.001
				2004	107, 732			
	**NPDR/PDR**	57	1.1	2000	88, 182	2.28 (2.22)	112, 779	0.042
				2004	200, 961			

Abbreviations: NPDR, non-proliferative diabetic retinopathy; PDR, proliferative diabetic retinopathy.

* Total cost & excess cost in NT dollars

**P value for t-test

### Changes in costs by progression of diabetic retinopathy

Table 2 also depicts the costs of medical care in NHI program enrollees in the two groups, PDR and NPDR. The presence and progression of diabetic retinopathy were associated with significant increases in costs of the NHI program. For example, average costs in the normal-to-normal category increased only NT$1,543 (US $48), from NT$62,218 (US $1921) in 2000 to NT$63,761 (US $1969) in 2004, whereas costs for those progressing from normal- to-NPDR or PDR increased NT$57,020 ($1760), from NT$50,712 (US $1566) in 2000 to NT$107,732 (US $3326) in 2004.

The normal category in both 2000 and 2004 had the lowest costs, and smaller relative increases in costs from 2000 to 2004. The PDR category had the highest average costs of NT$117,630 (US $3632) in 2000, but the NPDR-to-PDR category experienced the greatest increase in costs ( NT$112,779, US $3482), from NT$88,182 (US $2723) in 2000 to NT$200,961 (US $6204) in 2004. Regarding the percentage of increased costs between 2000 and 2004, the normal-to-NPDR-or-PDR category was 2.1% (2.1, adjusted by comparing to the normal category) and the NPDR-to-PDR category was 2.3% (adjusted to 2.2%), which were relatively higher than those in the stable group (Table 2).

## Discussion

Our current study is a longitudinal study that aimed to provide cost estimates associated with different severities of diabetic retinopathy in Taiwan. The number of medication-using diabetic subjects ≥ 40 years of age was 4,988 under NHI coverage in 2000. Of these 4,988 diabetic subjects, 82.5% were in the stable category, and 17.5% were in the progressive category during the 4-year follow-up. The normal category had the lowest costs and smaller relative increases in costs. The PDR category had the highest average costs in 2000, but the NPDR-to-PDR category experienced the greatest increase in costs from 2000 to 2004.

Any analysis of claims data is subject to significant limitations that have been well defined in the literature [16,17]. However, few studies have been published regarding the reliability and validity of such secondary data. In assessing diagnoses of diabetes, Lin et al. found that the accuracy of NHI claims data was 74.6% [18]. Variations in estimating the costs of diabetes care might result from alternative definitions for selecting diagnoses or combinations of diagnoses [19].

In Taiwan, the direct costs of healthcare for diabetic patients was 11.5% of the total national healthcare costs and was 4.3 times higher than the average cost of care for non-diabetic individuals from 1997 to 1998 [20].

In our study, diabetic subjects with diabetic retinopathy in 2000 had higher 2000 costs than those diabetic subjects with a normal retinal status in a cross-sectional view, but the normal-to-normal category had smaller relative increases in costs from 2000 to 2004 in a longitudinal view. One cross-sectional study conducted in Germany in 2002 also revealed that costs associated with diabetic retinopathy tend to increase as diabetic retinopathy progresses, being highest in patients with proliferative diabetic retinopathy and lowest in patients with mild, non-proliferative diabetic retinopathy [21]. One study conducted in the United States demonstrated that only part of the substantial expenditures associated with diabetic retinopathy, and with PDR in particular, are due to ophthalmic care [22].

In our study, subjects with proliferative diabetic retinopathy had the highest average costs in both 2000 and 2004, and subjects who progressed from NPDR to PDR experienced the greatest increases in costs. This evidence implies that strategies aimed at preventing and mitigating diabetic complications such as diabetic retinopathy are likely to be relative to long-term reductions in medical costs.

We acknowledge several limitations in our study. First, our analysis only included costs of medical care incurred by the NHI program in Taiwan. Second, we did not adjust for co-morbid diseases, and just focused on the relationship between overall medical costs and severities of diabetic retinopathy. Third, technological changes and economic inflation constitute a dynamic process. Diagnosis and treatment in 2000 may be somewhat different than that in 2004 as well as into the future, and cost escalation due to inflation may also be a factor. Both the results of absolute excess costs and relative percentage of increased costs should be taken into consideration. Fourth, misclassification bias in both the diagnosis and the grading of diabetic retinopathy should be considered when using claims data.

## Conclusions

In conclusion, we provide nationwide, large-scale, and longitudinal evidence that increased healthcare costs are associated with progressive diabetic retinopathy among diabetic NHI enrollees in Taiwan. It implies that strategies aimed at preventing and mitigating diabetic complications such as diabetic retinopathy are likely to be relative to long-term reductions in medical costs.

## Competing interests

The authors declare that they have no competing interests.

## Authors' contributions

PC and WHT participated in the design of the study and performed the statistical analysis. LCW and CYT have made substantial contributions to the study concept and design, or acquisition of data, or analysis and interpretation of data. HKC, MTT and STS conceived of the study, and helped to draft the manuscript or revise it critically for important intellectual content. All authors have read and approved the final manuscript.

## Pre-publication history

The pre-publication history for this paper can be accessed here:

http://www.biomedcentral.com/1472-6963/10/136/prepub
